# Association between psychological resilience and global health status/quality of life in patients with brain metastases after resection of primary non-small cell lung cancer

**DOI:** 10.3389/fpsyt.2026.1890412

**Published:** 2026-08-25

**Authors:** Caijun Dong, Yafen Jiang

**Affiliations:** 1 Department of Thoracic Surgery, Ningbo Second Hospital, Ningbo, Zhejiang, China; 2 Department of Neurology, Ningbo Second Hospital, Ningbo, Zhejiang, China

**Keywords:** brain metastases, non-small cell lung cancer, patient-reported outcomes, psychological resilience, quality of life

## Abstract

**Background:**

Brain metastases after resection of primary non-small cell lung cancer (NSCLC) impose substantial burdens. We examined the concurrent association between psychological resilience and global health status/quality of life (GHS/QoL).

**Methods:**

This single-center retrospective study included 207 adults who first developed brain metastases after primary NSCLC resection. Records from the 25-item Connor-Davidson Resilience Scale (CD-RISC), European Organisation for Research and Treatment of Cancer Quality of Life Questionnaire Core 30 (EORTC QLQ-C30), and European Organisation for Research and Treatment of Cancer Quality of Life Questionnaire Brain Neoplasm Module 20 (EORTC QLQ-BN20), collected at the same clinically stable outpatient assessment 4–12 weeks after brain-metastasis diagnosis, were linked to retrospectively extracted clinical data. Pearson correlations and a prespecified full-covariate multivariable linear regression model were used.

**Results:**

CD-RISC scores correlated positively with all QLQ-C30 functioning scales and GHS/QoL and negatively with the three QLQ-C30 symptom scales and four QLQ-BN20 multi-item symptom scales (all Benjamini–Hochberg-adjusted *q *< 0.001). Each 1-point increase in CD-RISC total score was associated with a 0.452-point higher adjusted GHS/QoL score (95% confidence interval, 0.326–0.578; *P *< 0.001). Higher Karnofsky Performance Status and college-level education were associated with higher GHS/QoL, whereas pretreatment clinical tumor-node-metastasis stage IIIB or IVA, ≥4 brain metastases, and 30-day complications after lung resection were associated with lower GHS/QoL.

**Conclusions:**

Greater psychological resilience was concurrently associated with better GHS/QoL and lower QLQ-BN20 multi-item symptom scores. These findings support integrated assessment of resilience, function, and symptoms; temporal direction requires longitudinal confirmation, and intervention effects require prospective trials.

## 1 Introduction

Lung cancer remains one of the malignancies contributing most heavily to global cancer incidence and mortality, with more than 2.4 million new cases worldwide in 2022 and a disease burden projected to continue increasing (1). Non-small cell lung cancer (NSCLC) accounts for most lung cancers, and its management now integrates surgery, radiotherapy, systemic therapy, and molecular profiling (2). Even after treatment of the primary tumor with curative intent, some patients with locally advanced NSCLC develop brain metastases during follow-up, underscoring the continuing clinical importance of surveillance for intracranial recurrence and subsequent multidisciplinary management (3). Brain metastases and their treatment may impair memory, attention, executive function, and activities of daily living, thereby complicating treatment decision-making, self-management, and social participation (4). Health-related quality of life integrates functional, symptomatic, and psychosocial burdens and is an important patient-reported outcome for supportive care, treatment trade-offs, and shared decision-making in patients with brain metastases (5). After diagnosis of brain metastases, quality of life is jointly influenced by neurological symptoms, functional status, treatment burden, and disease course, with substantial interindividual heterogeneity (6). Patients and their families often cope with disease uncertainty through information seeking, maintenance of daily routines, family collaboration, and meaning-making, yet some supportive needs may remain unrecognized (7). Symptom burden, illness perception, social support, and psychological resilience after lung cancer resection are interrelated, suggesting that psychological adaptation resources may vary alongside patient-reported outcomes (8). In patients with advanced cancer, greater psychological resilience commonly coexists with better quality of life, although social support, spirituality, and disease context may modify the strength of this association (9). Psychological resilience is a dynamic psychological resource that enables individuals to maintain or regain emotional and behavioral adaptation under substantial stress (10). This study aimed to describe psychological resilience and health-related quality of life among patients who first developed brain metastases after resection of primary NSCLC and to examine the concurrent association between the total score on the 25-item Connor-Davidson Resilience Scale (CD-RISC) and the global health status/quality of life (GHS/QoL) scale of the European Organisation for Research and Treatment of Cancer Quality of Life Questionnaire Core 30 (EORTC QLQ-C30).

## 2 Materials and methods

### 2.1 Study design and ethics

This single-center retrospective study was organized and reported in accordance with the Strengthening the Reporting of Observational Studies in Epidemiology (STROBE) statement (11). Using electronic medical records and an existing patient-reported outcomes database, we retrospectively and consecutively screened patients treated at Ningbo Second Hospital from March 2020 to December 2024 who first developed brain metastases after resection of primary non-small cell lung cancer (NSCLC). The included records from the 25-item Connor-Davidson Resilience Scale (CD-RISC), the European Organisation for Research and Treatment of Cancer Quality of Life Questionnaire Core 30 (EORTC QLQ-C30), and the European Organisation for Research and Treatment of Cancer Quality of Life Questionnaire Brain Neoplasm Module 20 (EORTC QLQ-BN20) originated from the same clinically stable outpatient assessment in the existing patient-reported outcomes database. Demographic characteristics, primary lung cancer surgery, pretreatment clinical tumor-node-metastasis (cTNM) stage, intracranial disease burden, functional status, and treatment exposures before questionnaire assessment were extracted from electronic medical records according to a prespecified data dictionary and linked to the questionnaire records. One investigator extracted the data, and a second investigator independently verified each item; discrepancies were resolved by reviewing the original medical records. The study was approved by the Ethics Committee of Ningbo Second Hospital (approval no. PJ-NBEY-KY-2026-195-01). Because this retrospective analysis used only existing electronic medical records and an existing patient-reported outcomes database, involved no additional intervention, examination, or new patient contact, and used data that had been de-identified before analysis, the ethics committee waived the requirement for written informed consent.

### 2.2 Participants

Patients were retrospectively and consecutively screened if they met all of the following criteria: (1) age ≥18 years; (2) pathologically confirmed NSCLC treated by resection of the primary lung cancer; (3) first occurrence of brain metastases during follow-up after lung resection, preferably confirmed by contrast-enhanced brain magnetic resonance imaging (MRI), or by contrast-enhanced computed tomography (CT) when MRI was contraindicated; (4) Karnofsky Performance Status (KPS) score ≥60; (5) estimated survival ≥3 months, as judged by the treating oncologist on the basis of current disease burden, organ function, and the treatment plan; (6) fulfillment of the prespecified cognitive and communication eligibility requirements and ability to complete the patient-reported outcome assessment; and (7) availability of prior questionnaire records and key clinical data. The KPS threshold of ≥60 and estimated survival of ≥3 months were used to ensure the basic functional capacity required to complete one patient-reported outcome assessment and to reduce the effect of immediate end-of-life clinical fluctuations on a single-time-point assessment; these criteria also define the population to which the findings apply.

Before questionnaire administration, patients were excluded for any of the following reasons: (1) another active malignancy; (2) severe cardiac, hepatic, renal, or other major organ dysfunction; (3) severe psychiatric disorder, cognitive impairment, aphasia, or another communication disorder that precluded completion of the patient-reported outcome assessment; (4) incomplete key clinical data; or (5) absence of a valid prior questionnaire assessment record or refusal to complete the prior outpatient questionnaire assessment. After questionnaire administration, quality control was performed on the basis of the computability of GHS/QoL, the computability of the CD-RISC total score, and the proportion of missing responses across the complete questionnaire set. The screening process was reported in three stages: exclusion before questionnaire assessment, completed assessment, and post-assessment quality control. Exclusion counts were assigned according to mutually exclusive primary reasons.

### 2.3 Staging and assessment of cognitive and communication abilities

Tumor-node-metastasis (TNM) staging of the primary lung cancer before initial treatment was recorded as clinical TNM (cTNM) stage and determined according to the eighth edition of the American Joint Committee on Cancer (AJCC)/Union for International Cancer Control (UICC) staging system (12). Stages IIIA, IIIB, and IVA refer to the pretreatment cTNM stage documented from imaging and clinical evaluation before initial treatment. All patients with stage IVA disease were highly selected clinical M1b (cM1b) cases who had no brain metastases at the time of lung resection; their M category was based on a single extracranial metastatic lesion. Lung resection was performed after multidisciplinary discussion confirmed that the primary lesion was resectable and that all known lesions were amenable to local treatment with curative intent. All patients were first diagnosed with brain metastases during follow-up after lung resection.

Cognitive ability was screened using the Chinese version of the Mini-Mental State Examination (MMSE), with education-adjusted study eligibility thresholds of ≥18 for patients without formal education, ≥20 for those with primary-school education, and ≥25 for those with junior-high-school education or above. These thresholds corresponded to cognitive-impairment cutoffs of MMSE <18, <20, and <25, respectively, used in a previous Chinese population study (13). Communication ability was assessed using a prespecified operational three-step task: restating the purpose of the assessment, accurately answering two questions about current health status, and clearly expressing a personal choice orally or in writing. The MMSE result and the three-step communication task were jointly used to determine whether the patient could complete the patient-reported outcome assessment.

### 2.4 Data collection and assessment timing

The following variables were collected retrospectively: sex, age, marital status, educational attainment, employment status, histological type, pretreatment cTNM stage of the primary lung cancer, number of brain metastases, maximum diameter of brain metastases, type of lung resection, complications within 30 days after lung resection, KPS score, and cumulative exposures to radiotherapy, chemotherapy, and targeted therapy from lung resection to questionnaire assessment. The three treatment categories could overlap, and combined exposure was described using mutually exclusive combinations. Radiotherapy included thoracic radiotherapy after lung resection or intracranial radiotherapy related to brain metastases; systemic treatment was categorized as chemotherapy or targeted therapy according to the medical record. The prior questionnaires were completed at the first clinically stable outpatient assessment 4–12 weeks after diagnosis of brain metastases. For intermittent radiotherapy or intravenous systemic therapy, the most recent course or cycle had ended at least 14 days before assessment and the next scheduled treatment had not begun; for ongoing oral targeted therapy, the dose had been stable for at least 14 days. At the time of assessment, no patient had acute treatment-related toxicity requiring urgent management. The intervals from lung resection to diagnosis of brain metastases, from lung resection to questionnaire assessment, from diagnosis of brain metastases to questionnaire assessment, and from the end of the most recent intermittent treatment to questionnaire assessment were recorded.

### 2.5 Questionnaire administration and quality control

Questionnaires were administered by uniformly trained research staff in a quiet outpatient assessment room. Patients completed them independently whenever possible. For patients unable to write because of visual impairment or restricted upper-limb movement, a staff member read each original item verbatim in a neutral manner and recorded the patient’s own response item by item, without explaining the item or prompting an answer; family members and caregivers did not answer by proxy. Missing items were checked when the questionnaires were returned, and the patient decided whether to provide the omitted response. An EORTC multi-item scale was scored when at least half of its component items had valid responses; in accordance with EORTC procedures, the score was calculated from the mean of the completed items. A missing single-item response remained missing. Calculation of the CD-RISC total score required valid responses to all 25 items. A questionnaire set was considered invalid if GHS/QoL could not be calculated, the CD-RISC total score could not be calculated, or more than 10% of the complete questionnaire set was missing. For the final 207 participants, the CD-RISC total score, all reported EORTC scale and single-item scores, and all covariates in the primary model were available. Complete-case analysis was used, without imputation across instruments.

### 2.6 Connor-Davidson resilience scale

Psychological resilience was assessed using the 25-item Chinese version of the Connor-Davidson Resilience Scale (CD-RISC). The scale comprises 13 tenacity items, 8 strength items, and 4 optimism items. Each item is scored from 0 to 4, yielding a total score of 0–100; higher scores indicate greater psychological resilience. This version has been used in Chinese patients with cancer and has shown good internal consistency (14). In the present sample, Cronbach’s *α* was 0.92 for the total scale and 0.86, 0.84, and 0.79 for the tenacity, strength, and optimism dimensions, respectively.

### 2.7 EORTC QLQ-C30

Health-related quality of life was assessed using the Chinese version of the EORTC QLQ-C30 (version 3.0). The questionnaire includes five functioning scales, three multi-item symptom scales, six single symptom items, and the GHS/QoL scale. Raw scores were linearly transformed to a 0–100 scale according to EORTC scoring procedures. Higher functioning-scale and GHS/QoL scores indicate better functioning or global health status/quality of life, whereas higher symptom-scale or single-item scores indicate greater symptom burden (15).

### 2.8 European organisation for research and treatment of cancer quality of life questionnaire brain neoplasm module 20

Brain-tumor-related symptoms were assessed using the Chinese version of the EORTC QLQ-BN20. The instrument comprises four multi-item symptom scales—future uncertainty, visual disorder, motor dysfunction, and communication deficit—and seven single items: headache, seizures, drowsiness, hair loss, itchy skin, weakness of legs, and bladder control (16).

All scale and item scores were transformed to a 0–100 scale; higher scores indicate more severe symptoms. International multilingual validation supports use of this module to assess health-related quality of life and symptoms in patients with brain tumors (17). Results were reported according to the established scoring structure as four multi-item symptom scales and seven single items.

### 2.9 Karnofsky performance status

The KPS score was independently rated by two trained clinicians on the basis of outpatient functional presentation and medical records; disagreements were resolved by discussion. Higher KPS scores are generally associated with better functioning and quality of life (18).

Clinical classification of the diagnosis and treatment of brain metastases was based on multidisciplinary guidelines for the management of brain metastases (19).

### 2.10 Statistical analysis

Analyses were performed using IBM SPSS Statistics for Windows, version 26.0 (IBM Corp., Armonk, NY, USA) and R version 4.4.2 (R Foundation for Statistical Computing, Vienna, Austria). Distributions of continuous variables were assessed using histograms, quantile-quantile (Q-Q) plots, and the Shapiro-Wilk test. Approximately normally distributed variables were summarized as mean ± standard deviation (SD), and skewed variables as median (interquartile range [IQR]). Categorical variables were summarized as counts and percentages. Continuous variables were compared between two groups using the independent-samples *t* test and among multiple groups using one-way analysis of variance. Homogeneity of variance was assessed with Levene’s test, with Welch correction applied when necessary. Categorical variables were compared using the *χ²* test or Fisher’s exact test. Comparisons of baseline characteristics were descriptive and exploratory, and unadjusted two-sided *P* values were reported. For multicategory variables, only overall between-group differences and mean trends were interpreted; these results were not taken to imply that every pairwise comparison differed. Linear associations between CD-RISC scores and quality-of-life scales were evaluated using Pearson correlation coefficients, and scatterplots were used to assess linearity. The 52 prespecified tests comprised combinations of four resilience scores with 13 multi-item scales or GHS/QoL, and the Benjamini-Hochberg procedure was used to control the false discovery rate.

The primary outcome was the QLQ-C30 GHS/QoL score. The primary analysis used a full-covariate multivariable linear regression model containing clinically relevant covariates, without selecting variables according to univariable *P* values. The model included 15 covariate constructs: CD-RISC total score, age, sex, marital status, educational attainment, employment status, pretreatment cTNM stage of the primary lung cancer, number of brain metastases, maximum diameter of brain metastases, 30-day complications after lung resection, KPS score, interval from diagnosis of brain metastases to questionnaire assessment, radiotherapy, chemotherapy, and targeted therapy. Educational attainment and pretreatment cTNM stage were each represented by two dummy variables, yielding 17 regression-coefficient degrees of freedom. Unstandardized regression coefficients (*B*), standard errors (SE), standardized regression coefficients (*β*), 95% confidence intervals (CI), *P* values, and variance inflation factors (VIF) were reported. Residual Q-Q plots and the Shapiro-Wilk test were used to assess residual normality, the Breusch-Pagan test to assess homoscedasticity, and partial residual plots to examine linearity of continuous variables. Each participant contributed one independent record. Cook’s distance was used to identify potentially influential observations, with 4/*n* prespecified as the sensitivity-screening threshold. Heteroskedasticity-consistent type 3 (HC3) robust standard errors and analyses excluding observations above this threshold were also reported. Stepwise regression was retained as an exploratory analysis. Sensitivity analyses included influential-observation analysis, exclusion of patients with pretreatment stage IVA disease, restriction to patients who completed the questionnaires independently, and an extended adjustment model additionally including histological type and type of lung resection. The primary outcome and all primary-model covariates were complete in the final analytical sample; no statistical imputation was performed. All tests were two-sided, with *α* = 0.05.

## 3 Results

### 3.1 Participant selection, assessment timing, and baseline characteristics

**Figure 1:**
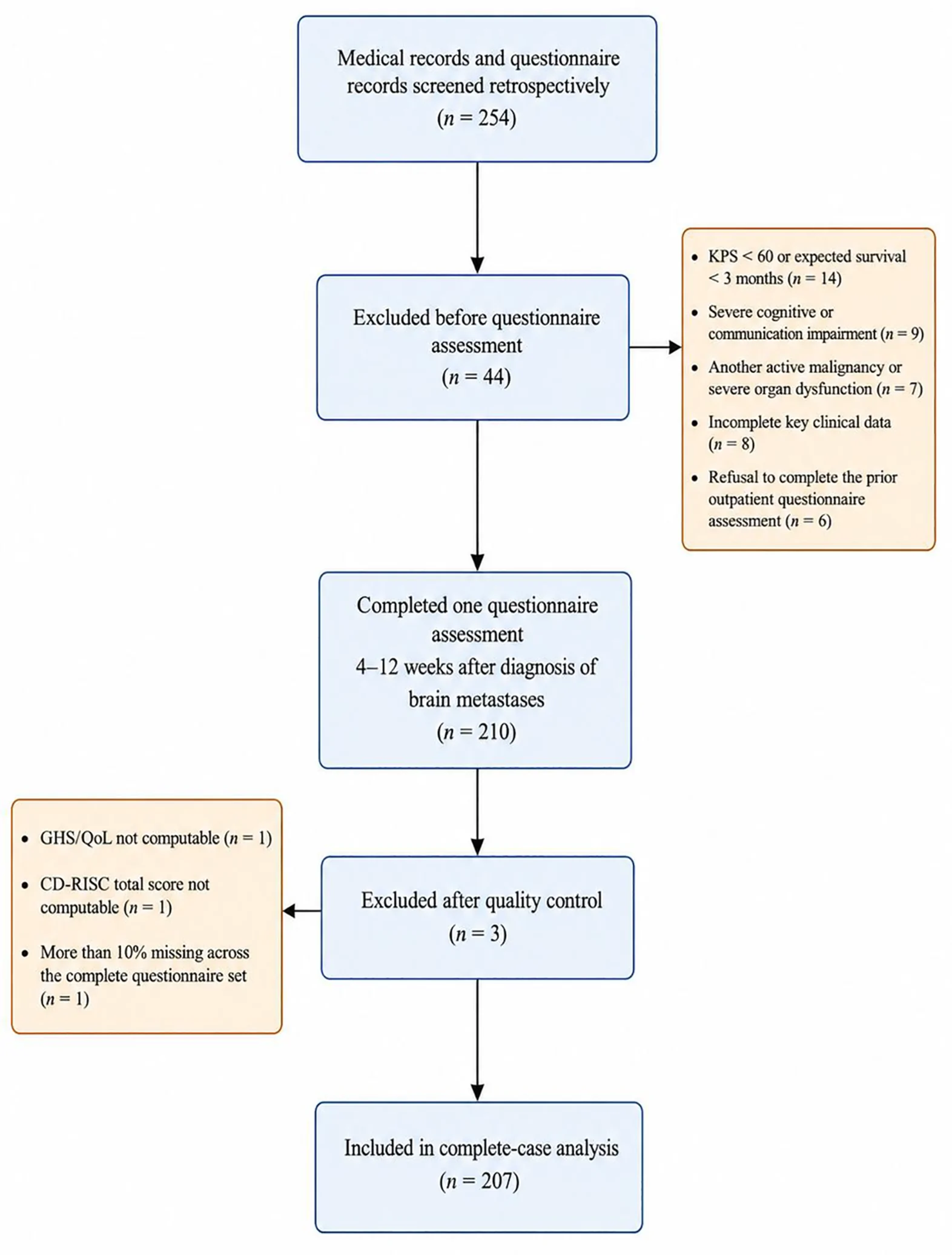
Participant selection and questionnaire-completion flowchart. Medical records and questionnaire records for 254 patients were screened retrospectively. Before questionnaire assessment, 44 patients were excluded according to mutually exclusive primary reasons, and 210 completed a single-time-point assessment 4–12 weeks after diagnosis of brain metastases. Quality control excluded three invalid questionnaire sets according to mutually exclusive primary reasons: global health status/quality of life (GHS/QoL) not computable (*n* = 1), Connor-Davidson Resilience Scale (CD-RISC) total score not computable (*n* = 1), and more than 10% missing across the complete questionnaire set (*n* = 1). A total of 207 participants entered the complete-case analysis. *n*, number of participants; IQR, interquartile range; KPS, Karnofsky Performance Status; QLQ-C30, Quality of Life Questionnaire Core 30; QLQ-BN20, Quality of Life Questionnaire Brain Neoplasm Module 20.

Medical records and questionnaire records for 254 patients were screened retrospectively. Before questionnaire assessment, 44 patients were excluded: KPS <60 or expected survival <3 months (*n* = 14), severe cognitive or communication impairment (*n* = 9), another active malignancy or severe organ dysfunction (*n* = 7), incomplete key clinical data (*n* = 8), and refusal to complete the prior outpatient questionnaire assessment (*n* = 6). A total of 210 patients completed one questionnaire assessment. Quality control excluded three questionnaire sets according to mutually exclusive primary reasons: GHS/QoL not computable (*n* = 1), CD-RISC total score not computable (*n* = 1), and more than 10% missing across the complete questionnaire set (*n* = 1). Thus, 207 participants were included in the complete-case analysis (**Figure 1**). Of these, 171 completed the questionnaires independently and 36 received neutral reading or writing assistance; all responses were provided by the patients themselves. The mean age was 61.24 ± 9.37 years (range, 36–78 years). The median intervals from lung resection to diagnosis of brain metastases, from lung resection to questionnaire assessment, and from diagnosis of brain metastases to questionnaire assessment were 18.2 months (IQR, 10.6–29.7), 19.8 months (IQR, 12.1–31.4), and 6.7 weeks (IQR, 5.1–8.6), respectively. Among the 199 patients who had received intermittent radiotherapy or intravenous systemic therapy before questionnaire assessment, the median interval from completion of the most recent course or cycle to questionnaire assessment was 19 days (IQR, 14–28).

The sample predominantly comprised men, patients aged ≥60 years, married patients, retired or unemployed patients, patients with adenocarcinoma, patients with 1–3 brain metastases, patients with a maximum brain-metastasis diameter <3 cm, patients who underwent lobectomy, patients without complications within 30 days after lung resection, and patients with KPS scores of 80–100. Pretreatment cTNM stages IIIA, IIIB, and IVA (cM1b) were similarly distributed. Cumulative exposure to radiotherapy and chemotherapy was common, whereas targeted therapy was less frequent. In descending order of frequency, the mutually exclusive treatment combinations were radiotherapy plus chemotherapy; radiotherapy plus chemotherapy plus targeted therapy; chemotherapy alone; targeted therapy alone; radiotherapy plus targeted therapy; chemotherapy plus targeted therapy; none of the three treatment categories; and radiotherapy alone. Younger age, married status, higher educational attainment, employment, earlier pretreatment cTNM stage, fewer brain metastases, absence of 30-day complications after lung resection, and higher KPS were associated with higher mean CD-RISC and GHS/QoL scores, with statistically significant overall between-group differences (all *P* < 0.05). Patients with a smaller maximum brain-metastasis diameter had a higher GHS/QoL score (*P* = 0.004), whereas the between-group difference in CD-RISC was not statistically significant (*P* = 0.204). Neither score differed significantly according to sex, histological type, type of lung resection, or exposure to radiotherapy, chemotherapy, or targeted therapy (all *P* > 0.05) (**Table 1**; **Supplementary Table 1**).

**Table 1:** Comparison of Connor-Davidson resilience scale (CD-RISC) and European organisation for research and treatment of cancer quality of life questionnaire core 30 (EORTC QLQ-C30) global health status/quality of life (GHS/QoL) scores according to patient characteristics (*n* = 207).

Characteristic	Category	*n* (%)	CD-RISC, mean ± SD	*t*/*F*	*P*	GHS/QoL, mean ± SD	*t*/*F*	*P*
Sex	Male	126 (60.9)	59.32 ± 15.69	0.687	0.493	53.13 ± 18.45	0.772	0.441
	Female	81 (39.1)	57.81 ± 15.03			51.12 ± 18.01		
Age, years	<60	89 (43.0)	62.43 ± 14.20	3.058	0.003	56.83 ± 17.36	3.134	0.002
	≥60	118 (57.0)	55.94 ± 15.77			48.96 ± 18.27		
Marital status	Married	173 (83.6)	60.19 ± 15.09	3.127	0.002	54.05 ± 17.78	3.105	0.002
	Unmarried/divorced/widowed	34 (16.4)	51.33 ± 15.17			43.63 ± 18.46		
Educational attainment	Junior high school or below	76 (36.7)	53.35 ± 14.81	9.910	<0.001	46.55 ± 17.64	8.325	<0.001
	High school/technical school	84 (40.6)	59.93 ± 14.57			53.48 ± 17.88		
	College or above	47 (22.7)	65.28 ± 15.16			59.67 ± 17.21		
Employment status	Employed	52 (25.1)	64.73 ± 14.79	3.319	0.001	59.69 ± 16.81	3.440	<0.001
	Retired/unemployed	155 (74.9)	56.72 ± 15.15			49.88 ± 18.11		
Histological type	Adenocarcinoma	148 (71.5)	59.51 ± 15.38	0.663	0.516	53.23 ± 18.16	0.630	0.533
	Squamous cell carcinoma	43 (20.8)	56.93 ± 15.74			50.34 ± 18.53		
	Other	16 (7.7)	56.40 ± 15.20			49.46 ± 18.91		
Pretreatment cTNM stage	IIIA	68 (32.9)	63.92 ± 14.21	7.900	<0.001	58.80 ± 16.71	8.553	<0.001
	IIIB	71 (34.3)	58.53 ± 14.86			51.93 ± 17.79		
	IVA (cM1b)	68 (32.9)	53.75 ± 15.68			46.32 ± 18.33		
Number of brain metastases	1–3	127 (61.4)	62.16 ± 14.73	4.189	<0.001	56.61 ± 17.06	4.428	<0.001
	≥4	80 (38.6)	53.29 ± 15.00			45.56 ± 18.14		
Maximum brain-metastasis diameter	<3 cm	142 (68.6)	59.65 ± 15.27	1.275	0.204	54.79 ± 17.61	2.907	0.004
	≥3 cm	65 (31.4)	56.71 ± 15.68			46.98 ± 18.64		
Type of lung resection	Lobectomy	154 (74.4)	59.29 ± 15.44	0.399	0.672	53.08 ± 18.27	0.495	0.611
	Pneumonectomy	38 (18.4)	56.96 ± 15.78			50.35 ± 18.59		
	Wedge resection	15 (7.2)	57.48 ± 14.94			49.80 ± 17.96		
30-day complications after lung resection	No	148 (71.5)	61.75 ± 14.52	4.676	<0.001	55.60 ± 17.32	4.237	<0.001
	Yes	59 (28.5)	51.17 ± 15.13			44.15 ± 18.13		
Radiotherapy before questionnaire assessment	Yes	179 (86.5)	59.13 ± 15.38	0.938	0.349	52.82 ± 18.16	0.948	0.344
	No	28 (13.5)	56.19 ± 15.72			49.30 ± 18.97		
Chemotherapy before questionnaire assessment	Yes	193 (93.2)	58.90 ± 15.48	0.587	0.558	52.58 ± 18.25	0.690	0.491
	No	14 (6.8)	56.39 ± 14.87			49.09 ± 18.78		
Targeted therapy before questionnaire assessment	Yes	89 (43.0)	60.14 ± 15.67	1.142	0.255	53.83 ± 17.94	1.018	0.310
	No	118 (57.0)	57.67 ± 15.21			51.22 ± 18.50		
KPS score	80–100	129 (62.3)	62.35 ± 14.80	4.549	<0.001	57.38 ± 17.04	5.450	<0.001
	60–70	78 (37.7)	52.74 ± 14.61			44.01 ± 17.21		

*n*, number of participants; %, percentage; CD-RISC, Connor-Davidson Resilience Scale; SD, standard deviation; *t*, independent-samples *t*-test statistic; *F*, one-way analysis-of-variance *F* statistic; *P*, two-sided *P* value; EORTC, European Organisation for Research and Treatment of Cancer; QLQ-C30, Quality of Life Questionnaire Core 30; GHS/QoL, global health status/quality of life; TNM, tumor-node-metastasis; cTNM, clinical TNM; AJCC, American Joint Committee on Cancer; UICC, Union for International Cancer Control; cM1b, clinical M1b; NSCLC, non-small cell lung cancer; KPS, Karnofsky Performance Status. Staging refers to pretreatment cTNM stage of the primary lung cancer according to the eighth edition of the AJCC/UICC system. All stage IVA cases were cM1b, had no brain metastases at lung resection, had an M category based on a single extracranial metastatic lesion, and received treatment with curative intent after multidisciplinary discussion. The “other” histological category comprised adenosquamous carcinoma (*n* = 7), large-cell carcinoma (*n* = 4), and NSCLC not otherwise specified (*n* = 5). Type of lung resection and 30-day complications refer to surgery for the primary lung cancer. Radiotherapy, chemotherapy, and targeted therapy represent cumulative exposures from lung resection to questionnaire assessment and may overlap. Two-group comparisons used the independent-samples *t* test, and multigroup comparisons used one-way analysis of variance. *P* values are unadjusted, two-sided, exploratory values.

### 3.2 Psychological resilience

The CD-RISC total score was used to describe overall psychological resilience. When the three dimensions were compared using mean item scores, optimism was highest, followed by tenacity and then strength. All dimensions and the total score were presented as continuous variables (**Table 2**).

**Table 2:** Connor-Davidson resilience scale (CD-RISC) dimension scores, total score, and mean item scores (*n* = 207).

Dimension	Number of items	Theoretical range	Raw score, mean ± SD	Mean item score, mean ± SD
Tenacity	13	0–52	30.45 ± 8.76	2.34 ± 0.67
Strength	8	0–32	18.62 ± 5.34	2.33 ± 0.67
Optimism	4	0–16	9.66 ± 3.18	2.42 ± 0.80
Total score	25	0–100	58.73 ± 15.42	2.35 ± 0.62

*n*, number of participants analyzed; CD-RISC, Connor-Davidson Resilience Scale; SD, standard deviation. The mean item score was calculated by dividing the raw dimension or total score by the corresponding number of items and was used for descriptive comparison across dimensions containing different numbers of items. Comparisons among dimensions were descriptive.

### 3.3 Health-related quality of life and brain-tumor-related symptoms

The QLQ-C30 functioning scales, from highest to lowest score, were physical, cognitive, role, emotional, and social functioning. The symptom scales, from highest to lowest score, were fatigue, pain, and nausea/vomiting; the single items, from highest to lowest score, were financial difficulties, insomnia, appetite loss, dyspnea, constipation, and diarrhea. GHS/QoL was presented as a separate scale. The QLQ-BN20 multi-item symptom scales, from highest to lowest score, were future uncertainty, motor dysfunction, visual disorder, and communication deficit; the single items, from highest to lowest score, were hair loss, drowsiness, weakness of legs, headache, itchy skin, bladder control, and seizures. Each score was interpreted according to the established direction of its scale (**Table 3**).

**Table 3:** European organisation for research and treatment of cancer quality of life questionnaire core 30 (EORTC QLQ-C30) and European organisation for research and treatment of cancer quality of life questionnaire brain neoplasm module 20 (EORTC QLQ-BN20) scale and item scores (*n* = 207).

Instrument	Category	Scale/item	Score, mean ± SD
EORTC QLQ-C30	Functioning scale	Physical functioning	64.32 ± 19.45
	Functioning scale	Role functioning	58.74 ± 21.38
	Functioning scale	Emotional functioning	55.27 ± 20.16
	Functioning scale	Cognitive functioning	60.83 ± 18.92
	Functioning scale	Social functioning	51.65 ± 22.74
	Symptom scale	Fatigue	45.73 ± 20.35
	Symptom scale	Pain	38.26 ± 22.18
	Symptom scale	Nausea/vomiting	25.84 ± 18.46
	Single item	Dyspnea	32.47 ± 21.59
	Single item	Insomnia	42.16 ± 24.73
	Single item	Appetite loss	36.95 ± 23.82
	Single item	Constipation	28.34 ± 20.67
	Single item	Diarrhea	18.52 ± 16.38
	Single item	Financial difficulties	51.68 ± 26.45
	Global scale	Global health status/quality of life	52.34 ± 18.26
EORTC QLQ-BN20	Multi-item symptom scale	Future uncertainty	58.47 ± 22.35
	Multi-item symptom scale	Visual disorder	35.62 ± 19.84
	Multi-item symptom scale	Motor dysfunction	42.18 ± 21.76
	Multi-item symptom scale	Communication deficit	31.25 ± 18.93
	Single item	Headache	38.74 ± 23.56
	Single item	Seizures	22.15 ± 17.42
	Single item	Drowsiness	46.83 ± 24.18
	Single item	Hair loss	52.36 ± 28.74
	Single item	Itchy skin	35.28 ± 21.47
	Single item	Weakness of legs	44.57 ± 22.89
	Single item	Bladder control	28.91 ± 19.65

*n*, number of participants analyzed; EORTC, European Organisation for Research and Treatment of Cancer; QLQ-C30, Quality of Life Questionnaire Core 30; QLQ-BN20, Quality of Life Questionnaire Brain Neoplasm Module 20; GHS/QoL, global health status/quality of life; SD, standard deviation. Higher QLQ-C30 functioning-scale and GHS/QoL scores indicate better functioning or global health status/quality of life. Higher QLQ-C30 symptom-scale, single-item, and QLQ-BN20 scores indicate more severe symptoms or problems. QLQ-BN20 results are presented as four multi-item symptom scales and seven single items.

### 3.4 Correlations between psychological resilience and quality of life

The CD-RISC total score and the tenacity, strength, and optimism dimension scores were positively correlated with QLQ-C30 physical, role, emotional, cognitive, and social functioning; negatively correlated with fatigue, pain, and nausea/vomiting; and positively correlated with GHS/QoL (all *P* < 0.001). The CD-RISC total score and all three dimension scores were negatively correlated with QLQ-BN20 future uncertainty, visual disorder, motor dysfunction, and communication deficit (all *P* < 0.001). All 52 prespecified correlation tests remained statistically significant after Benjamini-Hochberg adjustment; single symptom items were reported descriptively (**Table 4**).

**Table 4:** Pearson correlations of Connor-Davidson resilience scale (CD-RISC) scores with European organisation for research and treatment of cancer quality of life questionnaire core 30 (EORTC QLQ-C30) and European organisation for research and treatment of cancer quality of life questionnaire brain neoplasm module 20 (EORTC QLQ-BN20) outcomes (*n* = 207).

Outcome category	Scale/item	Tenacity	Strength	Optimism	CD-RISC total score
EORTC QLQ-C30 functioning scale	Physical functioning	0.512	0.476	0.398	0.536
	Role functioning	0.483	0.452	0.387	0.502
	Emotional functioning	0.536	0.498	0.425	0.567
	Cognitive functioning	0.465	0.437	0.371	0.485
	Social functioning	0.412	0.389	0.346	0.428
EORTC QLQ-C30 symptom scale	Fatigue	−0.438	−0.407	−0.352	−0.459
	Pain	−0.367	−0.342	−0.298	−0.382
	Nausea/vomiting	−0.305	−0.284	−0.247	−0.315
EORTC QLQ-C30 global scale	Global health status/quality of life	0.594	0.552	0.467	0.625
EORTC QLQ-BN20 multi-item symptom scale	Future uncertainty	−0.526	−0.487	−0.418	−0.548
	Visual disorder	−0.358	−0.332	−0.289	−0.372
	Motor dysfunction	−0.425	−0.394	−0.341	−0.442
	Communication deficit	−0.386	−0.358	−0.312	−0.401

*n*, number of participants analyzed; CD-RISC, Connor-Davidson Resilience Scale; EORTC, European Organisation for Research and Treatment of Cancer; QLQ-C30, Quality of Life Questionnaire Core 30; QLQ-BN20, Quality of Life Questionnaire Brain Neoplasm Module 20; GHS/QoL, global health status/quality of life; *r*, Pearson correlation coefficient; *P*, unadjusted two-sided *P* value; *q*, *P* value adjusted using the Benjamini-Hochberg (BH) procedure. The 52 tests comprised 4 resilience scores × 13 prespecified multi-item scales or GHS/QoL outcomes. All two-sided *P* values were <0.001, and all BH-adjusted *q* values were <0.001.

### 3.5 Multivariable analysis of GHS/QoL

**Figure 2:**
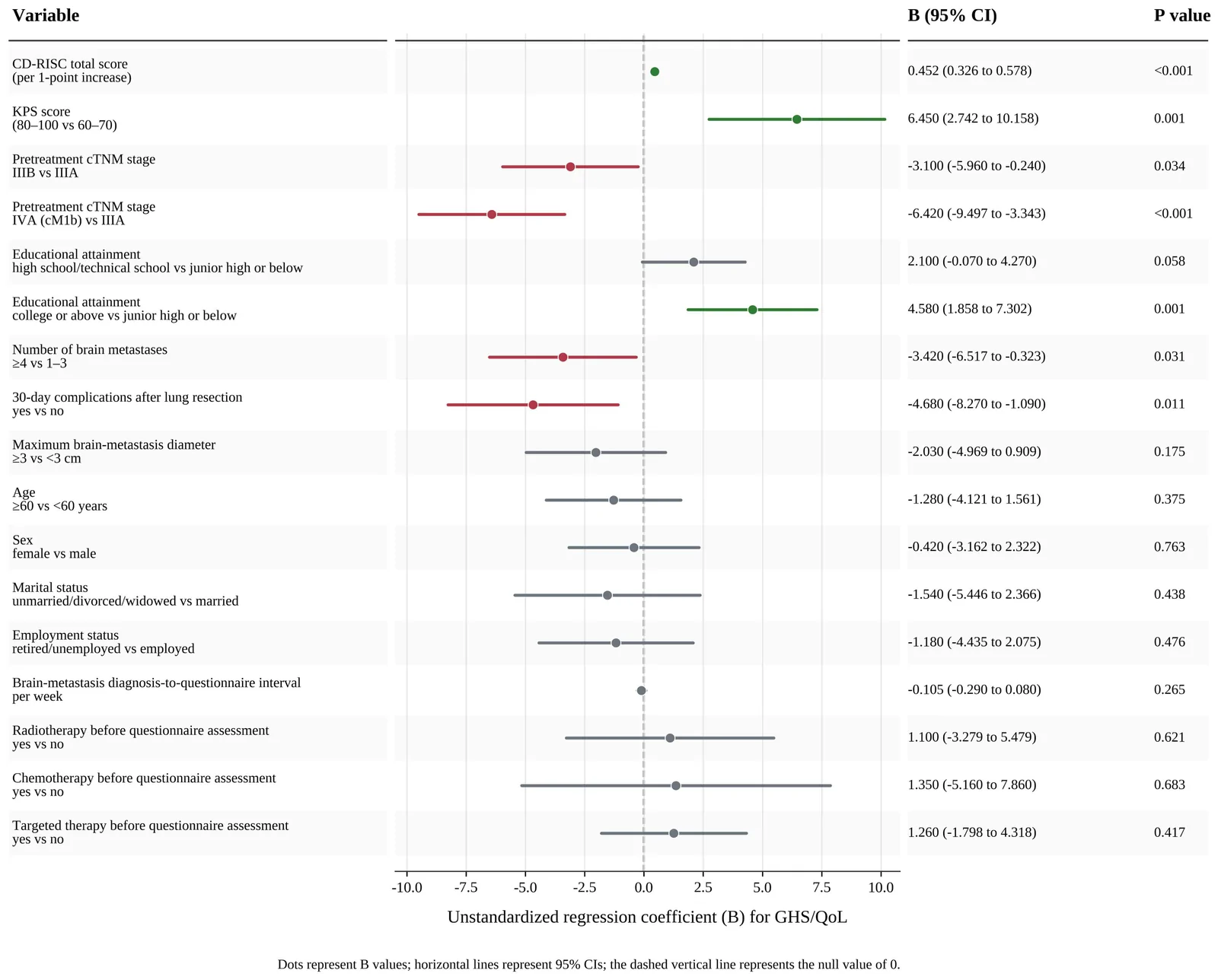
Fully adjusted multivariable linear regression model for the European Organisation for Research and Treatment of Cancer Quality of Life Questionnaire Core 30 (EORTC QLQ-C30) global health status/quality of life (GHS/QoL). Dots represent unstandardized regression coefficients (*B*), horizontal lines represent 95% confidence intervals (CI), and the vertical line represents the null value of 0. Reference categories for categorical variables are shown in the y-axis labels. The model contained 15 covariate constructs; educational attainment and pretreatment clinical tumor-node-metastasis (cTNM) stage of the primary lung cancer were each represented by two dummy variables, yielding 17 coefficient degrees of freedom. All pretreatment stage IVA cases were clinical M1b (cM1b). CD-RISC, Connor-Davidson Resilience Scale; KPS, Karnofsky Performance Status; *P*, two-sided *P* value; vs, versus.

In the full-covariate model, the CD-RISC total score showed a positive adjusted association with GHS/QoL (*P* < 0.001), and its standardized regression coefficient *β* had the largest absolute value among the model variables. Higher KPS and college-level education or above were associated with higher GHS/QoL, whereas pretreatment cTNM stage IIIB or IVA (cM1b), ≥4 brain metastases, and 30-day complications after lung resection were associated with lower GHS/QoL (all *P* < 0.05). The adjusted difference for high-school or technical-school education did not reach statistical significance (*P* = 0.058). Age, sex, marital status, employment status, maximum brain-metastasis diameter, interval from diagnosis of brain metastases to questionnaire assessment, radiotherapy, chemotherapy, and targeted therapy showed no statistically significant adjusted associations (all *P* > 0.05). Residuals were approximately normal (Shapiro-Wilk *W* = 0.992, *P* = 0.332), no evident heteroskedasticity was detected (Breusch-Pagan *χ²* = 15.84, *df* = 17, *P* = 0.535), and VIF values ranged from 1.18 to 1.95. Four observations had Cook’s distance >4/*n*; the maximum was 0.079, and all values were <1. Exploratory stepwise regression retained the CD-RISC total score, KPS, pretreatment stage IVA disease, college-level education or above, ≥4 brain metastases, and 30-day complications after lung resection; the directions of association were consistent with the full-covariate model (all *P* < 0.05). HC3 robust standard errors, exclusion of influential observations, exclusion of patients with pretreatment stage IVA disease, restriction to patients who completed the questionnaires independently, and an extended model additionally including histological type and type of lung resection all produced CD-RISC estimates of similar direction and magnitude (all *P* < 0.001) (**Figure 2**, **Table 5**; **Supplementary Tables 2**, **3**).

**Table 5:** Fully adjusted multivariable linear regression model for European organisation for research and treatment of cancer quality of life questionnaire core 30 (EORTC QLQ-C30) global health status/quality of life (GHS/QoL) (*n* = 207).

Variable	*B*	SE	Standardized *β*	*t*	95% CI	*P*	VIF
CD-RISC total score (per 1-point increase)	0.452	0.064	0.382	7.063	0.326 to 0.578	<0.001	1.89
KPS score (80–100 vs 60–70)	6.450	1.880	0.171	3.431	2.742 to 10.158	0.001	1.78
Pretreatment cTNM stage: IIIB vs IIIA	−3.100	1.450	−0.081	−2.138	−5.960 to −0.240	0.034	1.72
Pretreatment cTNM stage: IVA (cM1b) vs IIIA	−6.420	1.560	−0.165	−4.115	−9.497 to −3.343	<0.001	1.84
Educational attainment: high school/technical school vs junior high or below	2.100	1.100	0.057	1.909	−0.070 to 4.270	0.058	1.61
Educational attainment: college or above vs junior high or below	4.580	1.380	0.106	3.319	1.858 to 7.302	0.001	1.69
Number of brain metastases (≥4 vs 1–3)	−3.420	1.570	−0.091	−2.178	−6.517 to −0.323	0.031	1.49
30-day complications after lung resection (yes vs no)	−4.680	1.820	−0.116	−2.571	−8.270 to −1.090	0.011	1.58
Maximum brain-metastasis diameter (≥3 vs <3 cm)	−2.030	1.490	−0.052	−1.362	−4.969 to 0.909	0.175	1.43
Age (≥60 vs <60 years)	−1.280	1.440	−0.035	−0.889	−4.121 to 1.561	0.375	1.65
Sex (female vs male)	−0.420	1.390	−0.011	−0.302	−3.162 to 2.322	0.763	1.24
Marital status (unmarried/divorced/widowed vs married)	−1.540	1.980	−0.032	−0.778	−5.446 to 2.366	0.438	1.38
Employment status (retired/unemployed vs employed)	−1.180	1.650	−0.028	−0.715	−4.435 to 2.075	0.476	1.95
Brain-metastasis diagnosis-to-questionnaire interval (per week)	−0.105	0.094	−0.014	−1.117	−0.290 to 0.080	0.265	1.20
Radiotherapy before questionnaire assessment (yes vs no)	1.100	2.220	0.020	0.495	−3.279 to 5.479	0.621	1.31
Chemotherapy before questionnaire assessment (yes vs no)	1.350	3.300	0.019	0.409	−5.160 to 7.860	0.683	1.18
Targeted therapy before questionnaire assessment (yes vs no)	1.260	1.550	0.034	0.813	−1.798 to 4.318	0.417	1.33

*n*, number of participants analyzed; *B*, unstandardized regression coefficient; SE, standard error; *β*, standardized regression coefficient; *t*, *t* statistic for the regression coefficient; CI, confidence interval; *P*, two-sided *P* value; VIF, variance inflation factor; *R²*, coefficient of determination; adjusted *R²*, coefficient of determination adjusted for the number of covariates; *F*, model *F* statistic; *df*, degrees of freedom; *W*, Shapiro-Wilk statistic; *χ²*, chi-square statistic; vs, versus; CD-RISC, Connor-Davidson Resilience Scale; EORTC, European Organisation for Research and Treatment of Cancer; QLQ-C30, Quality of Life Questionnaire Core 30; GHS/QoL, global health status/quality of life; KPS, Karnofsky Performance Status; TNM, tumor-node-metastasis; cTNM, clinical TNM; cM1b, clinical M1b. *R²* = 0.635, adjusted *R²* = 0.602, *F*(17, 189) = 19.342, *P* < 0.001. The model contained 15 covariate constructs and yielded 17 coefficient degrees of freedom. Residual Shapiro-Wilk *W* = 0.992, *P* = 0.332; Breusch-Pagan *χ²* = 15.84, *df* = 17, *P* = 0.535; VIF range, 1.18–1.95. Partial residual plots supported approximate linearity of continuous variables. Each participant contributed one independent record. Four observations had Cook’s distance >4/*n*; the maximum was 0.079, and all were <1. Robustness analyses are presented in **Supplementary Table 3**.

## 4 Discussion

Among patients who first developed brain metastases after resection of primary NSCLC, psychological resilience showed a positive concurrent association with GHS/QoL and negative associations with brain-tumor-related multi-item symptom scores. These associations remained after adjustment for demographic characteristics, pretreatment cTNM stage of the primary lung cancer, intracranial disease burden, functional status, treatment exposures, 30-day complications after lung resection, and assessment timing. KPS, pretreatment cTNM stage, educational attainment, number of brain metastases, and 30-day complications after lung resection were also associated with GHS/QoL.

The positive association between psychological resilience and quality of life was directionally consistent with recent evidence in cancer populations, suggesting that goal maintenance, adaptive recovery, and problem-focused coping resources may co-occur with better patient-reported outcomes (20). Social support and coping style are associated with quality of life and psychological status in lung cancer, and support from families and healthcare teams may contribute to the link between resilience and quality of life; these potential pathways were not measured concurrently in this study (21). Illness perception and psychological resilience may be interrelated: patients’ understanding of their disease can shape adaptation, whereas functional and symptom status may alter how they appraise stress-related resources, providing a bidirectional interpretation of the concurrent association observed here (22). Stigma and self-perceived burden are associated with poorer quality of life and may jointly contribute, with resilience, to patients’ subjective experience of disease-related stress; subsequent studies should measure these variables within a prespecified mediation framework (23). Psychological resilience is context dependent and may change with disease course, treatment response, and everyday stress; repeated measurement may distinguish relatively stable individual differences from time-varying adaptation (24). Fatigue, pain, and nausea/vomiting were all negatively correlated with psychological resilience. Longitudinal studies could concurrently assess anxiety, depression, and coping styles to examine temporal pathways among symptom burden, psychological adaptation resources, and quality of life (25). Quality of life and cognitive outcomes in patients with brain metastases are simultaneously influenced by disease burden, radiotherapy, pharmacological treatment, and baseline neurocognitive status; psychosocial assessment should therefore be integrated with neurological function, treatment toxicity, and patient-reported symptoms (26). Future uncertainty had the highest score among the QLQ-BN20 multi-item symptom scales, suggesting that clinical communication may focus on treatment goals, anticipated symptoms, follow-up arrangements, and available support resources; individualized health education could be evaluated as a component of prospective supportive-care programs (27). The association of fatigue with quality of life may also be shaped by sense of coherence and stigma; in addition to symptom severity, assessment may consider patients’ interpretation of symptoms, perceived burden, and functional limitations (28). Higher educational attainment was associated with better GHS/QoL and may reflect differences in access to health information, communication efficiency, and the ability to use resources. Communication for patients with lower educational attainment could use plain-language materials, teach-back, and layered information delivery to improve accessibility (29). Subjective well-being among patients with brain tumors is associated with functional limitations, financial pressure, social support, and the care environment, consistent with the prominent descriptive burdens of financial difficulty and impaired social functioning in our sample (30). Psychosocial factors, symptoms, and functional status are jointly associated with quality of life in patients with brain tumors; multidisciplinary assessment can integrate patient-reported outcomes with neurological, oncological, and social support needs (31). Psychological burden and quality of life after lung cancer resection may vary across follow-up; repeated assessment can identify patients with persistent high burden or newly emerging support needs and provide temporal validation of the concurrent associations observed here (32). Cognitive-behavioral strategies, mindfulness practice, problem-solving training, and supportive counseling may be candidate psychological support modules for prospective trials. Programs could incorporate patient preferences, family involvement, digital follow-up, and symptom-triggered referral, with prespecified adherence, acceptability, GHS/QoL, and symptom outcomes (33). The associations of pretreatment cTNM stage, number of brain metastases, and KPS with GHS/QoL are consistent with the central role of disease burden and functional status in brain-metastasis management; future models should prespecify these clinical variables as adjustment factors (34). Lung cancer-related fatigue, dyspnea, appetite loss, and other symptoms may jointly limit functioning and quality of life, supporting a symptom-cluster approach to supportive care that incorporates psychological adaptation resources into comprehensive assessment (35). Each 1-SD increase in the CD-RISC total score corresponded to an approximately 6.97-point higher GHS/QoL score, representing the magnitude of the concurrent between-person association. The minimally important difference for the QLQ-C30 is primarily used to interpret within-person longitudinal change; the clinical meaning of this single-time-point between-person comparison requires further definition through repeated measurement and anchor-based analyses (36).

This study has several limitations. First, the retrospective design combined with a single-time-point questionnaire assessment can identify only concurrent covariation; temporal sequence, reverse causality, and within-person change require repeated measurement. Second, the study was conducted at a single center and included only patients with KPS ≥60, expected survival ≥3 months, and the ability to complete patient-reported outcome assessments. In addition, pretreatment stage IVA cases were restricted to cM1b patients selected by a multidisciplinary team for lung resection. Consequently, the burden among patients with more severe functional impairment or without surgical options may have been underestimated, limiting external validity. Third, historical surgical, staging, and treatment data were extracted from electronic medical records rather than patient recall. A prespecified data dictionary and independent verification by two investigators may have reduced abstraction and data-entry errors; however, incomplete documentation, variation in clinicians’ estimates of expected survival, and misclassification remain possible. Fourth, questionnaires relied on patient self-report, and neutral reading or writing assistance may have introduced administration-mode differences; however, sensitivity analysis restricted to independent completion produced similar findings. Fifth, questionnaires were administered 4–12 weeks after diagnosis of brain metastases and outside periods of acute treatment, but variation in treatment, symptoms, and disease status within this window still created residual temporal heterogeneity. Sixth, treatment variables were modeled as cumulative binary exposures. Specific regimens, doses, treatment timing, intracranial local therapy, and immunotherapy were not fully modeled; corticosteroid dose, molecular subtype, and extracranial disease burden were not systematically collected. Residual confounding by indication therefore remains possible. Seventh, anxiety, depression, coping style, social support, and health literacy were not measured concurrently; psychological pathways involving these variables require longitudinal models. Eighth, language, cultural context, and response conventions associated with the Chinese versions of the instruments may affect cross-cultural comparisons. Ninth, the primary model included 15 covariate constructs and yielded 17 coefficient degrees of freedom. Although residual diagnostics, VIF values, and multiple sensitivity analyses supported estimate stability, model performance requires validation in independent multicenter samples. Tenth, age, number of brain metastases, and maximum lesion diameter were categorized using prespecified cutoffs, potentially causing information loss; larger samples could support continuous or nonlinear modeling.

## 5 Conclusions

Among patients who first developed brain metastases after resection of primary NSCLC, greater psychological resilience was associated with better QLQ-C30 GHS/QoL and lower QLQ-BN20 multi-item symptom scores. KPS, pretreatment cTNM stage of the primary lung cancer, educational attainment, number of brain metastases, and 30-day complications after lung resection were also associated with GHS/QoL. These concurrent associations support integrated assessment of psychological adaptation resources, functional status, and symptom burden; temporal direction and the effects of supportive strategies require confirmation in multicenter longitudinal studies and prospective trials.

## Data Availability

The original contributions presented in the study are included in the article/**Supplementary Material**. Further inquiries can be directed to the corresponding author.

## Supplementary material

Supplementary Table 1 Mutually exclusive combinations of radiotherapy, chemotherapy, and targeted therapy before questionnaire assessment (*n* = 207). Note: *n*, number of participants; %, percentage. Each mutually exclusive combination was classified according to actual cumulative exposure between lung resection and questionnaire assessment. Based on the combination counts, the marginal numbers exposed to radiotherapy, chemotherapy, and targeted therapy were 179, 193, and 89, respectively; 199 patients had received either radiotherapy or chemotherapy. Percentages may not sum to 100.0% because of rounding. Treatment exposures were nonrandom cumulative binary variables; specific regimens and timing are addressed in the study limitations.

Supplementary Table 2 Exploratory stepwise regression model for European Organisation for Research and Treatment of Cancer Quality of Life Questionnaire Core 30 (EORTC QLQ-C30) global health status/quality of life (GHS/QoL) (*n* = 207). Note: *n*, number of participants analyzed; *B*, unstandardized regression coefficient; SE, standard error; *β*, standardized regression coefficient; *t*, *t* statistic for the regression coefficient; CI, confidence interval; *P*, two-sided *P* value; VIF, variance inflation factor; *R²*, coefficient of determination; adjusted *R²*, coefficient of determination adjusted for the number of covariates; *F*, model *F* statistic; *df*, degrees of freedom; vs, versus; CD-RISC, Connor-Davidson Resilience Scale; EORTC, European Organisation for Research and Treatment of Cancer; QLQ-C30, Quality of Life Questionnaire Core 30; GHS/QoL, global health status/quality of life; KPS, Karnofsky Performance Status; TNM, tumor-node-metastasis; cTNM, clinical TNM; cM1b, clinical M1b. This model was an exploratory sensitivity analysis; the full-covariate model was the primary basis for interpretation. *R²* = 0.616, adjusted *R²* = 0.604, *F*(6, 200) = 53.472, *P* < 0.001.

Supplementary Table 3 Robustness and extended-adjustment sensitivity analyses of the association between the Connor-Davidson Resilience Scale (CD-RISC) and global health status/quality of life (GHS/QoL). Note: *n*, number of participants analyzed; *B*, unstandardized regression coefficient for the association between the CD-RISC total score and GHS/QoL; SE, standard error; CI, confidence interval; *P*, two-sided *P* value; HC3, heteroskedasticity-consistent type 3 robust standard error; CD-RISC, Connor-Davidson Resilience Scale; GHS/QoL, global health status/quality of life; cM1b, clinical M1b; Cook’s distance, a diagnostic measure of the influence of an individual observation on regression estimates. Four observations had Cook’s distance >4/*n*; the maximum was 0.079, and all were <1. Analyses using HC3 robust standard errors, excluding these four observations, excluding patients with pretreatment stage IVA (cM1b) disease, restricting the sample to patients who completed the questionnaires independently, and further adjusting for histological type and type of lung resection all retained the same direction and a similar effect magnitude.
