# Supplementary material for: Association between psychological resilience and global health status/quality of life in patients with brain metastases after resection of primary non-small cell lung cancer
Source: Front Psychiatry. 2026 Aug 25;17:1890412. doi: 10.3389/fpsyt.2026.1890412 (PMC13550854; doi:10.3389/fpsyt.2026.1890412)
Supplement: Supplementary file 1 [file Supplementaryfile1.docx]

**Supplementary Material**

**Supplementary Table 1. Mutually exclusive combinations of radiotherapy, chemotherapy, and targeted therapy before questionnaire assessment (*n* = 207)**

| **Mutually exclusive treatment combination** | ***n*** | **%** |
| --- | --- | --- |
| Radiotherapy + chemotherapy | 97 | 46.9 |
| Radiotherapy + chemotherapy + targeted therapy | 76 | 36.7 |
| Chemotherapy alone | 16 | 7.7 |
| Targeted therapy alone | 5 | 2.4 |
| Radiotherapy + targeted therapy | 4 | 1.9 |
| Chemotherapy + targeted therapy | 4 | 1.9 |
| None of the three treatment categories | 3 | 1.4 |
| Radiotherapy alone | 2 | 1.0 |
| Total | 207 | 100.0 |

**Note:** *n*, number of participants; %, percentage. Each mutually exclusive combination was classified according to actual cumulative exposure between lung resection and questionnaire assessment. Based on the combination counts, the marginal numbers exposed to radiotherapy, chemotherapy, and targeted therapy were 179, 193, and 89, respectively; 199 patients had received either radiotherapy or chemotherapy. Percentages may not sum to 100.0% because of rounding. Treatment exposures were nonrandom cumulative binary variables; specific regimens and timing are addressed in the study limitations.

**Supplementary Table 2. Exploratory stepwise regression model for European Organisation for Research and Treatment of Cancer Quality of Life Questionnaire Core 30 (EORTC QLQ-C30) global health status/quality of life (GHS/QoL) (*n* = 207)**

| **Variable** | ***B*** | **SE** | **Standardized *β*** | ***t*** | **95% CI** | ***P*** | **VIF** |
| --- | --- | --- | --- | --- | --- | --- | --- |
| CD-RISC total score (per 1-point increase) | 0.478 | 0.059 | 0.404 | 8.102 | 0.362 to 0.594 | <0.001 | 1.61 |
| KPS score (80–100 vs 60–70) | 7.220 | 1.740 | 0.191 | 4.149 | 3.789 to 10.651 | <0.001 | 1.47 |
| Pretreatment cTNM stage: IVA (cM1b) vs IIIA | −6.010 | 1.420 | −0.154 | −4.232 | −8.810 to −3.210 | <0.001 | 1.39 |
| Educational attainment: college or above vs junior high or below | 4.210 | 1.240 | 0.098 | 3.395 | 1.765 to 6.655 | 0.001 | 1.32 |
| Number of brain metastases (≥4 vs 1–3) | −3.800 | 1.480 | −0.101 | −2.568 | −6.718 to −0.882 | 0.011 | 1.28 |
| 30-day complications after lung resection (yes vs no) | −5.210 | 1.700 | −0.129 | −3.065 | −8.562 to −1.858 | 0.002 | 1.36 |

**Note:** *n*, number of participants analyzed; *B*, unstandardized regression coefficient; SE, standard error; *β*, standardized regression coefficient; *t*, *t* statistic for the regression coefficient; CI, confidence interval; *P*, two-sided *P* value; VIF, variance inflation factor; *R²*, coefficient of determination; adjusted *R²*, coefficient of determination adjusted for the number of covariates; *F*, model *F* statistic; *df*, degrees of freedom; vs, versus; CD-RISC, Connor-Davidson Resilience Scale; EORTC, European Organisation for Research and Treatment of Cancer; QLQ-C30, Quality of Life Questionnaire Core 30; GHS/QoL, global health status/quality of life; KPS, Karnofsky Performance Status; TNM, tumor-node-metastasis; cTNM, clinical TNM; cM1b, clinical M1b. This model was an exploratory sensitivity analysis; the full-covariate model was the primary basis for interpretation. *R²* = 0.616, adjusted *R²* = 0.604, *F*(6, 200) = 53.472, *P* < 0.001.

**Supplementary Table 3. Robustness and extended-adjustment sensitivity analyses of the association between the Connor-Davidson Resilience Scale (CD-RISC) and global health status/quality of life (GHS/QoL)**

| **Analysis** | ***n*** | ***B*** | **SE** | **95% CI** | ***P*** |
| --- | --- | --- | --- | --- | --- |
| Primary full-covariate model | 207 | 0.452 | 0.064 | 0.326 to 0.578 | <0.001 |
| HC3 robust standard errors | 207 | 0.452 | 0.067 | 0.320 to 0.584 | <0.001 |
| Excluding 4 observations with Cook’s distance >4/*n* | 203 | 0.448 | 0.062 | 0.326 to 0.570 | <0.001 |
| Excluding patients with pretreatment stage IVA (cM1b) disease | 139 | 0.439 | 0.079 | 0.283 to 0.595 | <0.001 |
| Patients who completed questionnaires independently only | 171 | 0.445 | 0.070 | 0.307 to 0.583 | <0.001 |
| Extended adjustment including histological type and type of lung resection | 207 | 0.449 | 0.065 | 0.321 to 0.577 | <0.001 |

**Note:** *n*, number of participants analyzed; *B*, unstandardized regression coefficient for the association between the CD-RISC total score and GHS/QoL; SE, standard error; CI, confidence interval; *P*, two-sided *P* value; HC3, heteroskedasticity-consistent type 3 robust standard error; CD-RISC, Connor-Davidson Resilience Scale; GHS/QoL, global health status/quality of life; cM1b, clinical M1b; Cook’s distance, a diagnostic measure of the influence of an individual observation on regression estimates. Four observations had Cook’s distance >4/*n*; the maximum was 0.079, and all were <1. Analyses using HC3 robust standard errors, excluding these four observations, excluding patients with pretreatment stage IVA (cM1b) disease, restricting the sample to patients who completed the questionnaires independently, and further adjusting for histological type and type of lung resection all retained the same direction and a similar effect magnitude.
